# The efficacy and safety of platelet-rich plasma in the tendon-exposed wounds: a preliminary study

**DOI:** 10.1186/s13018-022-03401-0

**Published:** 2022-11-19

**Authors:** Zhuan Deng, Zhi-Sheng Long, Fei-Peng Gong, Gang Chen

**Affiliations:** 1 Department of Orthopedics, Jiangxi Provincial People’s Hospital, The First Affiliated Hospital of Nanchang Medical College, Nanchang, 330006 Jiangxi China; 2 Medical College, Nanchang University, Nanchang, 330006 Jiangxi China

**Keywords:** Platelet-rich plasma, Tendon-exposed wounds, Refractory wound, Skin flap transplantation

## Abstract

**Objective:**

Currently, among wounds with large skin tissue defects caused by various reasons, the treatment of refractory wounds is still a major clinical problem. This study is aimed to preliminarily assess the therapeutic potentials of platelet-rich plasma (PRP) in refractory wounds with exposed tendons, as well as corresponding efficacy and safety.

**Methods:**

A total of 12 patients (5 males and 7 females) with refractory wounds and exposed tendons who were admitted to our hospital from June 2018 to December 2020 were included in this study. After the preparation of PRP, the included patients underwent the PRP injection after the debridement of wounds, and the efficacy and prognosis were assessed by the same group of senior surgeons.

**Results:**

The average age of included patients was 42.7 ± 12.9 years, and the causes of injury included traffic accidents (3 cases), contusion (2 cases), burns (2 cases), diabetes complications (4 cases), and melanoma complications (1 cases). The average healing time was 23.0 ± 5.0 days, and the mean size of the wound was 3.1 × 5.1 cm^2^. During the whole treatment process, Vancouver Scar Scale (VSS) decreased from 7.4 ± 1.6 before PRP treatment to 3.6 ± 0.9 after treatment (*P* < 0.001), Manchester Scar Scale (MSS) decreased from 12.3 ± 4.5 before PRP treatment to 5.4 ± 1.2 after treatment (*P* < 0.001), and no redness and swelling were observed around wounds, the size and degree of wounds gradually reduced, the coverage rate of granulation tissue was acceptable, overall quality of scar was relatively good, skin sensitivity around wounds was normal, there was no local wounds secretion, and postoperative patient's satisfaction was relatively good during follow-up.

**Conclusions:**

Our study has preliminarily indicated that PRP can promote the wounds healing, reduce the inflammation around wounds, and improve the granulation tissue and angiogenesis, thereby effectively polishing up the safety and efficacy.

## Introduction

With the rapid development of transportation and construction industries, the skin and subcutaneous soft tissue defects are more common in the clinic [1, 2]. It is well known that when the tissue defect is small, the body can generally heal itself [3, 4]. However, if the trauma is large, it may result in the exposure of vital tissue deep under the skin, such as tendons or bones. Currently, among the wounds with large skin tissue defects caused by a variety of reasons, the treatment of refractory wounds is still a major clinical problem, which requires stringent and advanced soft tissue management technologies [5, 6]. Especially when the wounds exist in the limbs that need to carry out the complicated life functions and social roles [7, 8]. Moreover, the coverage of soft tissue defects in limbs involves several techniques ranging from the simple second-intention healing to skin grafting with or without dermal substitutes, to local homodigital or heterodigital flaps and partial toe transfers, and the arsenal in terms of skin coverage and especially flaps is diverse [9, 10]. Thus, successful and timely treatment and management are significant [11–13]. Otherwise, it is easy to produce a series of complications, such as wounds infection, scar contracture, and dysfunction of functional activity at wound sites, and serious cases may eventually result in disability, which might cause huge psychological and economic burden to patients and reduce the quality of life [14, 15].

The healing of body wounds is a complicated and dynamic process [16]. Notably, conventional treatment methods are to remove necrotic tissue after skin debridement and then select an appropriate part of the body for skin flap transplantation to cover this kind of refractory wounds [17, 18]. However, wound healing is hindered by reduced activity or quantity of local growth factors at the tissue defect sites and poor vascularity in exposed areas of bone or tendon [19], and the damage of normal skin tissue caused by flap transplantation is often difficult for patients to undertake. With regard to this, it is worth noting that the recent evidence of regenerative biomaterial platelet-rich plasma (PRP) in the treatment of such refractory wounds has been widely recognized [20].

PRP is a therapeutic biological product extracted from the venous blood of the body, which contains high concentrations of platelets [21]. Platelet particles contain a super mixture of cytokines and several growth factors, such as platelet-derived growth factor (PDGF), transforming growth factor-β (TGF-β), epidermal growth factor (EGF), fibroblast growth factor (FGF), vascular endothelial growth factor (VEGF), and so on [22]. This mixture can promote the angiogenesis and repair in the partial tissue defect sites, which is also the key point to promote the wound angiogenesis and bright red granulation tissue formation. Therefore, the application of PRP has attracted extensive attention in the fields of repair and regenerative medicine and has broad application prospects and research value. On the basis of this, this study is aimed to preliminarily evaluate the therapeutic potentials of PRP in such refractory wounds with exposed tendons, as well as corresponding efficacy and safety, so as to provide a reference for broader clinical applications in the future.

## Methods and materials

### Patients

This study was approved by the Ethics Review Committee of Jiangxi Provincial People's Hospital, The First Affiliated Hospital of Nanchang Medical College, and all patients signed an informed consent to participate in the study. A total of 12 patients (5 males and 7 females) with refractory wounds and exposed tendons were admitted to Jiangxi Provincial People’s Hospital, The First Affiliated Hospital of Nanchang Medical College from June 2018 to December 2020. The inclusion criteria mainly included: (1) 18–65 years old and no gender restrictions; (2) injuries that mainly lied in extremities and caused by various reasons, such as trauma, contusion, burns, diabetes complications, and so on; and (3) all patients who participated voluntarily and signed the informed consent. The exclusion criteria mainly included: (1) the color Doppler ultrasound of blood vessels that showed severe lesions of large blood vessels with obvious ischemia; (2) the exposed area of wound that was too large or combined with exposed bone, blood vessels, and other wounds that must be repaired with skin flaps; (3) complicated with severe dysfunction of other important organs; (4) combined with malignant tumors; and (5) the patients who received glucocorticoids, immunosuppressive agents, and chemotherapy. Moreover, refractory wounds mainly refer to conditions that ulcers are still difficult to improve, the secretion is still not reduced, and the granulation tissue is not obviously formed after the applications of repeated dressing changes, negative pressure vacuum suction, and local application of various drugs [12, 16].

### Preparation of PRP

During the extraction process of PRP (PRP kit, WEGO Medical Instruments Ltd., Weihai, Shandong, China), the first step of centrifugation was mainly to separate the red blood cells (RBCs), and the second step of centrifugation was to obtain concentrated platelets. Specifically, a catheter containing acid citrate dextrose (ACD) anticoagulant was applied to collect the venous blood of the patients. Next, a 10-min centrifugation was performed via an inclined centrifuge with a rotating speed of 2500 rpm. Then, the obtained centrifugal solution was mainly divided into the following three layers: (1) the upper layer was the serum layer, and the content of platelets was very rare; (2) the middle layer was the platelet-rich plasma layer, which was rich in platelets; (3) the bottom layer was mainly composed of the RBCs. Subsequently, the upper layer of serum and the middle layer of platelets were transferred to a new empty sterile tube to perform the second centrifugation, and the upper layer of obtained centrifugation (composed of platelet-deficient plasma) was removed, thereby retaining the lower layer to obtain the PRP.

### Debridement of wounds and injection of PRP

**Fig. 1:**
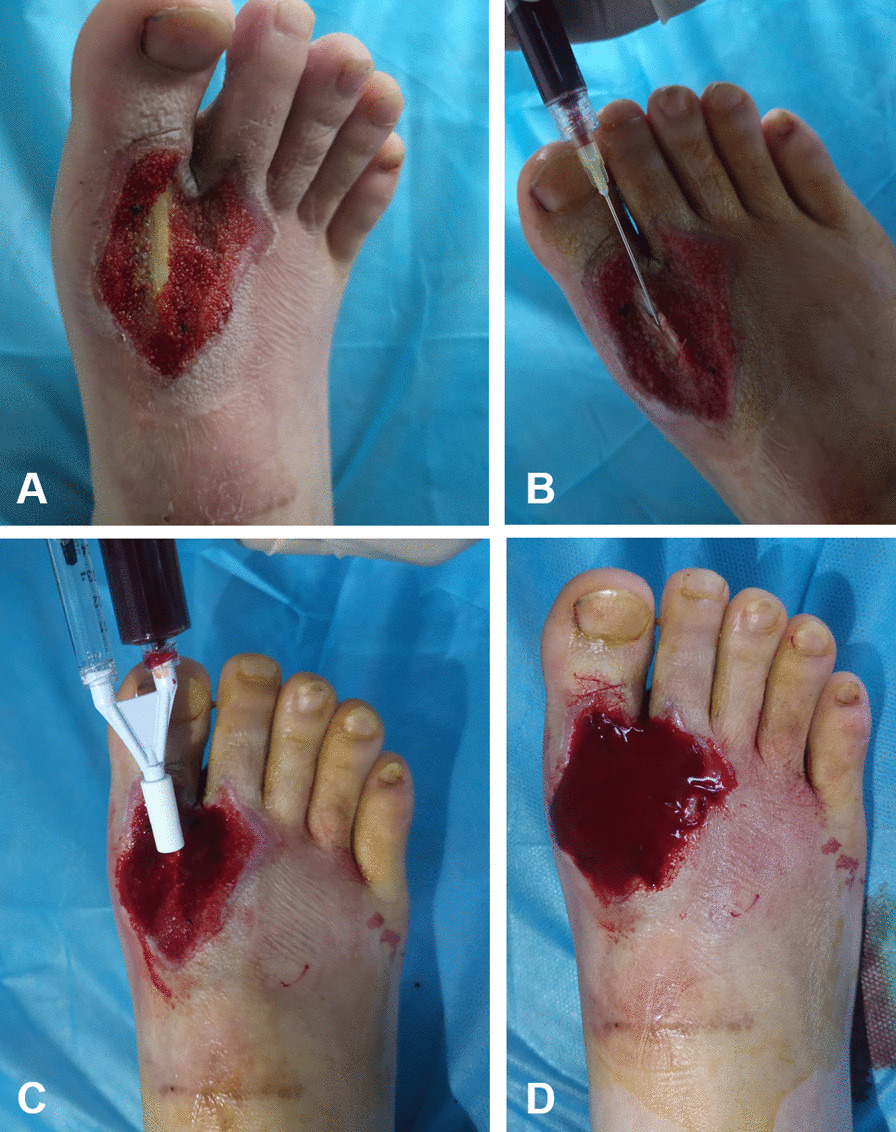
Specific steps of the injection of PRP in refractory wounds with exposed tendons. **A** The appearance of refractory wounds with exposed tendons; **B**–**D** The injection of PRP in refractory wounds with exposed tendons

The wounds debridement and preparation of PRP should be conducted simultaneously, and all the operations of wounds debridement were performed in the operating room by the same group of senior surgeons. In brief, the wounds were fully exposed and covered with sterile gauze, and the wound surface was thoroughly disinfected with iodophor. After the onset of local anesthesia, the wound surfaces were repeatedly irrigated with normal saline, hydrogen peroxide, and iodophor, and the necrotic tissues, purulent secretions, and inflammatory attachments were completely removed at the same time. Next, the wounds were cleaned repeatedly until it was judged by senior doctors that it was suitable for clinical injection. Therein, the criteria for judging whether it can be sutured is to judge bacterial contents (infection degree) according to the degree of inflammation around the wounds and the amount and viscosity of secretions [23]. Subsequently, PRP was extracted with a syringe and evenly covered on the wounds, an appropriate dressing was selected for low tension dressing, and routine dressing changes were performed once a day after the operation. The specific steps of the operation are shown in Fig. 1. In addition to this, due to the half-life of platelets being estimated to be 5 to 7 days, on the 7th day after the operation, the same operation was performed again [24].

### Evaluation criteria

During the whole treatment period, the wounds conditions of all included patients were assessed by the same group of senior doctors. The evaluation indicators mainly included the changes in appearance and color around wounds, changes in wound size and depth, the pain level of wounds, wounds healing degree, local wounds secretion, granulation tissue coverage, scar quality, wounds healing time, postoperative patients’ satisfaction with wound appearance, and so on. Therein, the pain level of the wounds was mainly assessed by Visual Analog Scale (VAS) [25]. The assessment of wound healing was primarily performed by visual observation and superficial measurements, such as monitoring the size, color, odor, drainage, and eschar of the wound, and the Vancouver Scar Scale (VSS) [26] and Manchester Scar Scale (MSS) [27] were used to quantify the wound assessment outcomes for patients. Specifically, each patient was evaluated by the surgeons from the same medical team before and after PRP treatment. Therein, the evaluation of VSS did not need special equipment, but only relied on the naked-eye observation of tester. The patient's scar was palpated with bare hands, and measured from four aspects: color, thickness, blood vessel distribution, and softness. The overall condition of scar was evaluated in the form of a scoring system, and the highest score was 18 points. Moreover, the evaluation of MSS on the scar was mainly from the next five aspects: scar surface, edge height, thickness, color, and chroma. The evaluation value of the color score in the scale was 13 points, and the evaluation value of other scores was 16 points. The higher the score, the more severe the scar.

### Statistical analysis

After performing the normality tests, the data (demographic and clinical data of included patients and clinical data on wounds) in this current study were exhibited as count (percentage) or mean ± standard deviation (SD) and were analyzed by paired *t* test for continuous variables in IBM SPSS Statistics for Windows, Version 23.0. (IBM Corp. Armonk, New York, United States). A *P* value < 0.05 was considered statistically significant.

## Results

### Demographic and clinical data

The demographic and clinical data of included 12 patients (5 males and 7 females) are exhibited in Table 1, and the clinical data on wounds are exhibited in Table 2. Therein, the average age of included patients was 42.7 ± 12.9 years, and the causes of injury mainly included traffic accidents (3 cases), contusion (2 cases), burns (2 cases), diabetes complications (4 cases) and melanoma complications (1 cases). The average healing time was 23.0 ± 5.0 days, and the mean size of the wound was 3.1 × 5.1 cm^2^. During the whole treatment process, the VSS decreased from 7.4 ± 1.6 before PRP treatment to 3.6 ± 0.9 after treatment (*P* < 0.001), the MSS decreased from 12.3 ± 4.5 before PRP treatment to 5.4 ± 1.2 after treatment (*P* < 0.001), and no redness and swelling were observed around the wounds, the size and degree of the wounds gradually reduced, the coverage rate of granulation tissue was acceptable, the overall quality of scar was relatively good, the skin sensitivity around wounds was normal, there was no local wounds secretion, and the postoperative patients' satisfaction was relatively good during the follow-up.

**Table 1:** Demographic and clinical data of included patients

Age	Gender	Injured part	Causes	Healing time(days)	Size of wound (cm^2^)
57	Male	Above the first metatarsal of the right foot	Traffic accident	18	2 × 3
24	Male	Middle of third metacarpal bone on the dorsal side of left hand	Contusion	25	3 × 5
28	Male	Lower anterior tibia of right leg	Burns	19	6 × 8
32	Male	Dorsal proximal carpal joint of right forearm	Traffic accident	17	2 × 5
45	Male	Nearly 2 cm behind the fourth toe of the left sole	Melanoma complications	29	2 × 2
42	Female	Outer front of right plantar	Diabetes complications	30	3 × 6
49	Female	The right plantar contains the ankle canal	Diabetes complications	30	2 × 5
52	Female	Medial malleolus and anterior malleolus of right foot	Diabetes complications	15	4 × 6
65	Female	Outer front of left plantar	Diabetes complications	21	3 × 5
27	Female	Lower 1/3 of medial tibia of right lower leg	Burns	27	5 × 7
35	Female	Dorsal proximal wrist of left forearm	Contusion	23	2 × 3
56	Female	Front middle of right forearm	Traffic accident	22	3 × 6

**Table 2:** Clinical data on wounds of included patients

	VAS	VSS	MSS
Before PRP treatment	6.9 ± 1.6	7.4 ± 1.6	12.3 ± 4.5
After PRP treatment	3.5 ± 0.7	3.6 ± 0.9	5.4 ± 1.2
*P* value	0.021	< 0.001	< 0.001

*PRP* platelet-rich plasma; *VAS* Visual Analog Scale; *VSS* Vancouver Scar Scale; *MSS* Manchester Scar Scale

### Typical case

A 56-year-old middle-aged female was hospitalized with pain and dysfunction of right forearm due to mechanical injury. Physical examination revealed a wound on the front of right forearm, about 5 cm × 4 cm in size, and the flexor tendon of forearm was exposed. During the operation, large wounds were found, the direct suture may have large tension, and the possibility of postoperative complications was high. Meanwhile, the patient disagreed with secondary trauma caused by autologous skin flap transplantation, and then surgeons communicated with the patient and her family to choose PRP therapeutic schedule, and they have also signed the informed consent.

**Fig. 2:**
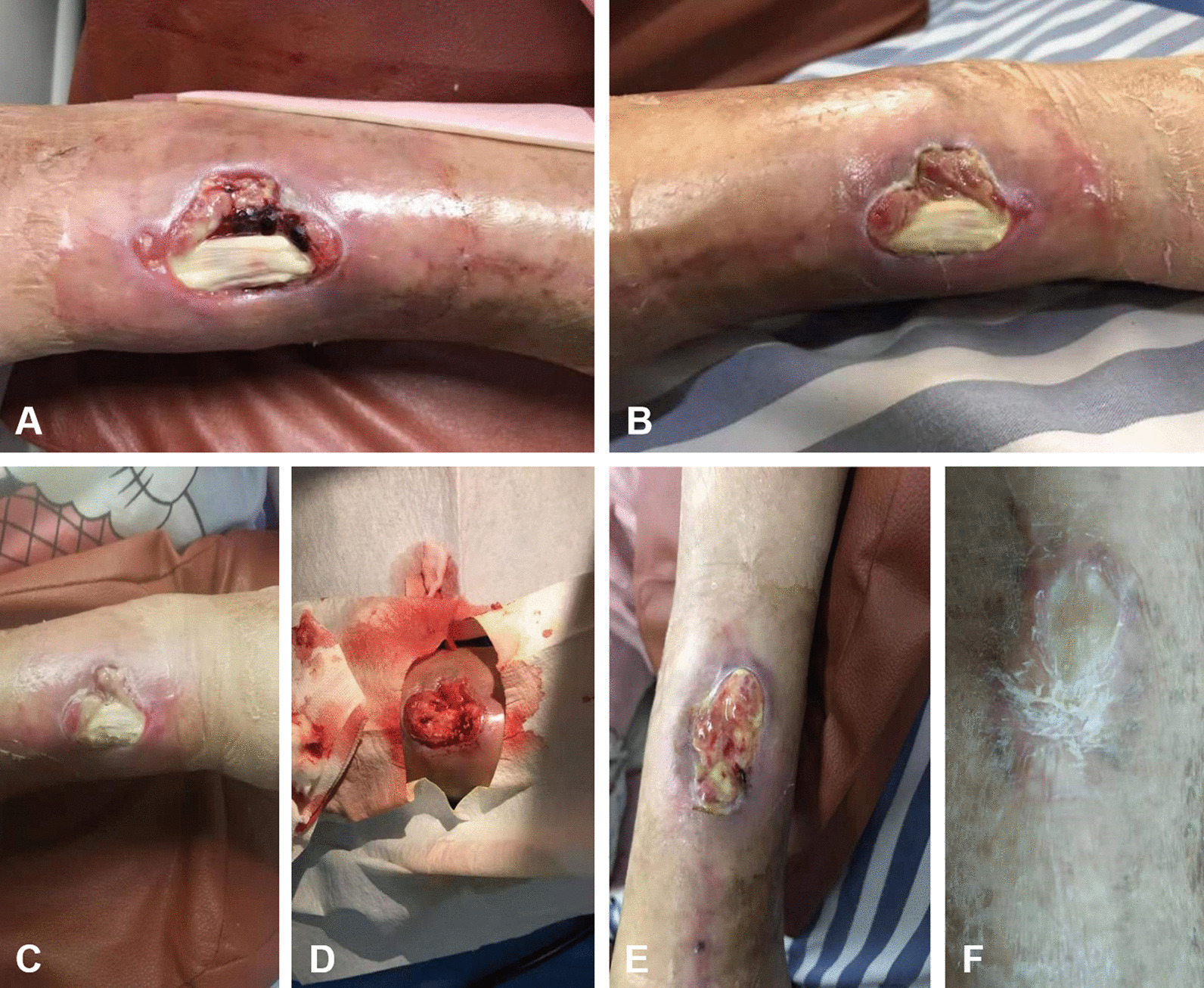
Situation and treatment process of the patient with refractory wounds with exposed tendons. **A** The growth of granulation tissues around tendon was found on the 4th day after the operation; **B** The granulation tissues covered all wounds except tendon on the 5th day; **C** The granulation tissue began to cover the partial tendon on the 6th day; **D** During the second operation, it was observed that the blood supply of the wounds was abundant; **E** The whole wounds were covered with granulation tissue, and the wound area reduced to 3.8 cm × 2.6 cm on the 5 days after the second operation; **F** The condition after wound healing

Subsequently, according to the above steps, the patient underwent the PRP operation. The wound pain was significantly relieved on the first day after the operation, and the VAS score dropped from 7 to 3. Moreover, the growth of granulation tissues around the tendon was observed on the 4th day after the operation (Fig. 2A). On the 5th day, the granulation tissues covered all wounds except tendon (Fig. 2B). On the 6th day, the granulation tissue began to cover the partial tendon (Fig. 2C). During this period, the wound area reduced to 4.1 cm × 3.5 cm, and there was no obvious inflammation around the wounds. Due to the half-life of platelets was estimated to be 5 to 7 days [24], on the 7th day after the operation, the same operation was performed again. During the second operation, it was observed that the blood supply of the wounds was abundant (Fig. 2D). On the 5 days after the second operation, the whole wounds were covered with granulation tissue, and the wound area reduced to 3.8 cm × 2.6 cm (Fig. 2E). Moreover, the wound pain also disappeared basically at this time. On the 22 days of the treatment cycle, the wounds healed. Throughout the treatment process, the patient underwent two surgeries, and the wounds area reduced to 3.6 cm × 2.3 cm eventually, good-quality scar and no obvious complications were found. Follow-ups at 1, 3, 6, and 12 months after discharge indicated no significant abnormalities.

## Discussion

Due to the characteristics of wide coverage, the complex pathogenesis, and the difficult treatment of chronic refractory wounds with exposed tendons, the cure rate is relatively low, and the treatment cycle is relatively long and the cost is relatively high, which directly influences the quality of life of patients [28]. The traditional skin transplantation and flap reconstruction have long been the main treatment methods for this kind of refractory wounds with the exposed tendons, while the success rate of flap transplantation and associated postoperative complications are still troublesome problems currently [4]. Especially when wounds exist in the parts with very shallow soft tissue, such as the inner front edge of tibia, medial, and lateral malleolus, wrist, and other parts of body, it is apparent that the success rate of flap transplantation in these parts is not optimistic [29, 30]. With regard to this, this study has preliminarily applied the PRP in the treatment of refractory wounds with the exposed tendons, and its efficacy and safety are relatively considerable. Specifically, in this current study, during the whole treatment process, VSS decreased from 7.4 ± 1.6 before PRP treatment to 3.6 ± 0.9 after treatment (*P* < 0.001), MSS decreased from 12.3 ± 4.5 before PRP treatment to 5.4 ± 1.2 after treatment (*P* < 0.001), and no redness and swelling were observed around wounds, size and degree of wounds gradually reduced, coverage rate of granulation tissue was acceptable, overall quality of scar was relatively good, skin sensitivity around wounds was normal, there was no local wounds secretion, and the postoperative patients’ satisfaction was relatively good during the follow-up.

PRP is a skin substitute that allows dermis-like tissue to grow over the stromal layer and vascularization in the areas of exposed bone or tendon, which provides a powerful adjunct option for the reconstructive surgeries [31]. Recently, it has been observed that the PRP can trigger the process of granulation formation and initiate healing [32]. Moreover, it is the formation of these vascular tissues that improves the microcirculation obstruction and increases local blood flow, while the newly formed vascular permeability is relatively high. The macrophages, monocytes, and neutrophils could also be chemotactic by PRP and gather at the wound to phagocytize necrotic tissues and cells, and promote wounds healing. In addition, providing an extracellular matrix state suitable for cell proliferation plays a vital role in the process of wounds healing [33]. A single growth factor has been used in the past to promote the wounds healing, with clear results and no effective repair [34]. In addition, it is acknowledged that there are various growth factors and various cytokines in PRP. When activated, it can release these bioactive substances to provide a suitable microenvironment for the tissues repair, thus affecting the process of wounds healing. Moreover, there are also reports of anti-inflammatory factors in partial previous studies, which strongly indicates that PRP also has anti-inflammatory effects. Similarly, there is also evidence that there are some antimicrobial peptides in substances released by PRP, and the presence of these peptides makes this biological product have a certain antibacterial effect [35]. Accordingly, the patients included in this current study also exhibited no signs of inflammation and infection during the process of treatment and prognosis.

Recently, PRP and other emerging tissue engineering technologies have gradually become popular alternatives for the field of reconstruction [36, 37]. Compared with the traditional treatment measures, PRP is more likely to cause a favorable clinical outcome, which can effectively treat the refractory wounds with exposed tendons, improve the aesthetic appearance, and significantly reduce the occurrence of complications at the trauma sites [38, 39]. On the other hand, simplicity, immediacy, safety, and abundant availability of this biomaterial are the important considerations in the application of PRP [40, 41]. It is known to us that the PRP is easy to obtain in the clinical practice and can effectively reduce the medical expenditure, and the curative effects of vascular regeneration are reliable, which is of particular clinical significance in the treatment operation required for exposed tendon coverage and can provide the material nutrition metabolism for the wounds. Furthermore, compared with flap transplantation, PRP is less technically demanding, which allows the operation to be conducted in several hospitals of different grades and provides the surgeons with a more practical treatment option.

In addition, Menchisheva et al. [42] reported that the application of PRP was beneficial for the early emergence of wounds granulation tissue and angiogenesis, which was consistent with the results of our study. In the current typical case, on the 3rd day after the first operation, the granulation tissue appeared, and the bright red particles almost covered the entire wounds on the 7th day. Behind these phenomena, partial scholars speculated that it might be related to the mechanisms that released growth factor binds to corresponding receptors of VEGF and basic FGF on cell membrane [43]. Then, through PI3K/Akt signaling pathway, the signal is transmitted downward step by step, and the signal is transmitted to the nucleus to promote the synthesis of related proteins. Moreover, it is also possible to promote the cell proliferation, matrix synthesis, and angiogenesis of normal endothelial cells. Guo et al. [44] revealed that both the Erk and Akt were phosphorylated, thereby activating the angiogenesis-related signaling pathways. Through these two significant pathways, local vascularization has been effectively promoted and then further promoted the formation of granulation tissue, thereby increasing the blood supply at the wound sites.

Ultimately, although recent studies have revealed that the efficacy and advantages of PRP are of great significance, we also need to consider the shortcomings of this current study. Firstly, there is no clear and unified view on the optimal concentration of PRP, and the platelet activity, the amount of various growth factors released by platelets, and the mixed leukocytes in different patients are also different, which has brought great trouble to the daily clinical work. Secondly, the associated molecular mechanisms of PRP promoting wounds healing are still not clear, which may easily result in the unstable prognosis of partial patients in the clinic. Thirdly, there are several versions of the methods for the extraction and activation of PRP currently, and the final efficacy is not able to be strictly and standard evaluated. In addition, the methods and processes for extracting, preserving, and applying PRP to refractory wounds need to be better explored and developed. As this study was a preliminary study, the sample size of reported cases was relatively small, and no adverse effect was reported during this period. We expect to include a larger sample size in the future researches, so as to conduct more complete and detailed studies. Collectively, these points still need to be verified in more standard and more rigorous prospective and large-sample trials in the future.

## Conclusions

To sum up, our current study has preliminarily revealed that PRP can promote wounds healing, reduce inflammation around wounds, and improve granulation tissue and angiogenesis, thus effectively polishing up the safety and clinical efficacy. PRP is expected to become a novel and potential treatment method for the refractory wounds with exposed tendons in the future.

## Data Availability

The datasets used and/or analyzed during this study are available from the corresponding author upon reasonable request.
